# Terminal 4q duplication and extended 10q deletion in a preterm infant with linear growth restriction: transcriptomic evidence of disrupted developmental and metabolic pathways

**DOI:** 10.3389/fped.2026.1850899

**Published:** 2026-06-18

**Authors:** Eva Teresa Töpfer, Marion Zähringer, Michael K. Baumgartner, Anne Ch. Garbe, Désirée Dunstheimer, Moneef Shoukier, Olena Karachun, Ulrike Walden, Cornelia Daumer-Haas, Michael C. Frühwald, Melanie L. Conrad, Victoria E. Fincke, Pascal D. Johann, Fabian B. Fahlbusch

**Affiliations:** 1 Neonatology and Pediatric Intensive Care, Faculty of Medicine, University of Augsburg, Augsburg, Germany; 2 Center for Rare Diseases Augsburg (AZeSE), Faculty of Medicine, University of Augsburg, Augsburg, Germany; 3 Eurofins Humangenetik und Pränatal-Medizin MVZ GmbH, München, Germany; 4 Pediatric Nephrology, Faculty of Medicine, University of Augsburg, Augsburg, Germany; 5 Swabian Children’s Cancer Center, Faculty of Medicine, University of Augsburg, Augsburg, Germany; 6 Comprehensive Cancer Center Augsburg (CCCA ), Faculty of Medicine, University of Augsburg, Augsburg, Germany

**Keywords:** 10q26 deletion, epithelial-mesenchymal signaling, gene dosage effect, terminal 4q duplication, transcriptome profiling

## Abstract

**Background:**

Distal duplication 4q syndrome and distal 10q26 deletion syndrome are rare chromosomal abnormalities associated with complex and overlapping phenotypes. Most cases result from unbalanced inheritance of a parental translocation, yet the specific contributions of each chromosomal segment to the clinical phenotype remain poorly defined.

**Case presentation:**

We report on a male preterm infant born at 28 + 6 weeks' gestation who carried a derivative chromosome 10 due to a paternally inherited unbalanced translocation t(4;10)(q31.22;q26.13), leading to a 42.46 Mb duplication of 4q31.22–q35.2 and a 10.77 Mb deletion of 10q26.13–q26.3. The patient presented with severe postnatal linear growth restriction, delayed neurodevelopment, a giant umbilical hernia, bilateral renal hypoplasia, and relative overweight. Thumb anomalies were absent.

**Methods:**

Genetic analyses included chromosomal microarray, conventional karyotyping, fluorescence *in situ* hybridization (FISH), and transcriptome profiling from peripheral blood mononuclear cells.

**Results:**

Gene expression analysis confirmed reduced expression of established 10q26-related genes, including *FGFR2*, *EMX2*, *WDR11*, *NSMCE4A*, and *EBF3*, and additionally identified reduced expression of *DMBT1*, *CUZD1*, *CTBP2*, *CHST15*, *OAT*, and *LHPP*. These genes are implicated in epithelial–mesenchymal signaling, extracellular matrix organization, developmental regulation, and metabolic pathways. Regional transcriptomic analysis further supported dosage-associated effects, with lower averaged expression across genes represented on the array within the deleted 10q26.13-10q26.3 interval and higher averaged expression across genes within the duplicated 4q31.22–4q35.2 interval compared with the reference dataset. Adjacent flanking regions did not show comparable directional shifts, supporting the interpretation that the observed transcriptional changes were regionally aligned with the structural chromosomal imbalance rather than reflecting a global PBMC expression difference.

**Conclusion:**

This case illustrates how extended terminal deletions of 10q can disrupt key structural and metabolic gene networks. To our knowledge, this is the first transcriptomic characterization of a 10.77 Mb deletion in the 10q26.13–q26.3 region together with a large terminal 4q duplication. Integrating functional transcriptomics with clinical and cytogenetic data may enhance our understanding of rare chromosomal disorders and inform individualized management, including reproductive counseling and longitudinal clinical follow-up.

## 1 Introduction

### 1.1 Cytogenetic background and previous reports

Trisomy 4q syndrome was first reported in 1971 (1), with more than 60 cases subsequently documented. However, distal trisomy 4q remains a rare finding (2). Chromosome 10q26 deletion syndrome was first described in a similar period (3) and has been documented in over 110 cases (4). To the best of our knowledge, only two previously published cases have described a comparable inherited unbalanced t(4;10) with partial trisomy of distal 4q and a small terminal 10q deletion (5, 6). In both reports, molecular analyses demonstrated large distal 4q copy-number gains involving 4q26–4q35.2, combined with small terminal 10q26.3 microdeletions. Zhang et al. mapped the 4q breakpoint to 4q26, between 115,596,658 and 118,785,802 bp, with an associated approximately 0.54-Mb 10q26.3 deletion. Popescu et al. reported a 71,057-kb copy-number gain of 4q26–4q35.2 at genomic coordinates 119,839,900–190,896,674 on chromosome 4, together with a 562-kb deletion of 10q26.3 at coordinates 134,872,533–135,434,178 on chromosome 10. Thus, both cases were dominated by a large distal 4q copy-number gain of approximately 71–72 Mb, whereas the accompanying 10q26.3 deletion was comparatively small, approximately 0.54–0.56 Mb. Accordingly, the trisomy 4q component was hypothesized to be the major contributor to the observed phenotype.

In general, 4q distal duplication is a heterogeneous disorder based on the multitude of breakpoints that are distributed throughout the long arm of chromosome 4 (excluding the 4q11 band). In a comprehensive overview of breakpoint regions and their associated phenotypes, Popescu et al. showed that proximal 4q partial trisomy regions (4q23–q27) were associated with milder phenotypes (5). In contrast, we present a case with a more distal 4q breakpoint, resulting in a 42.46 Mb terminal duplication of 4q31.22–q35.2. Notably, the accompanying 10q deletion was substantially larger than in previously reported cases, measuring 10.77 Mb (5, 6).

### 1.2 Clinical synopsis and phenotypic features

**Figure 1:**
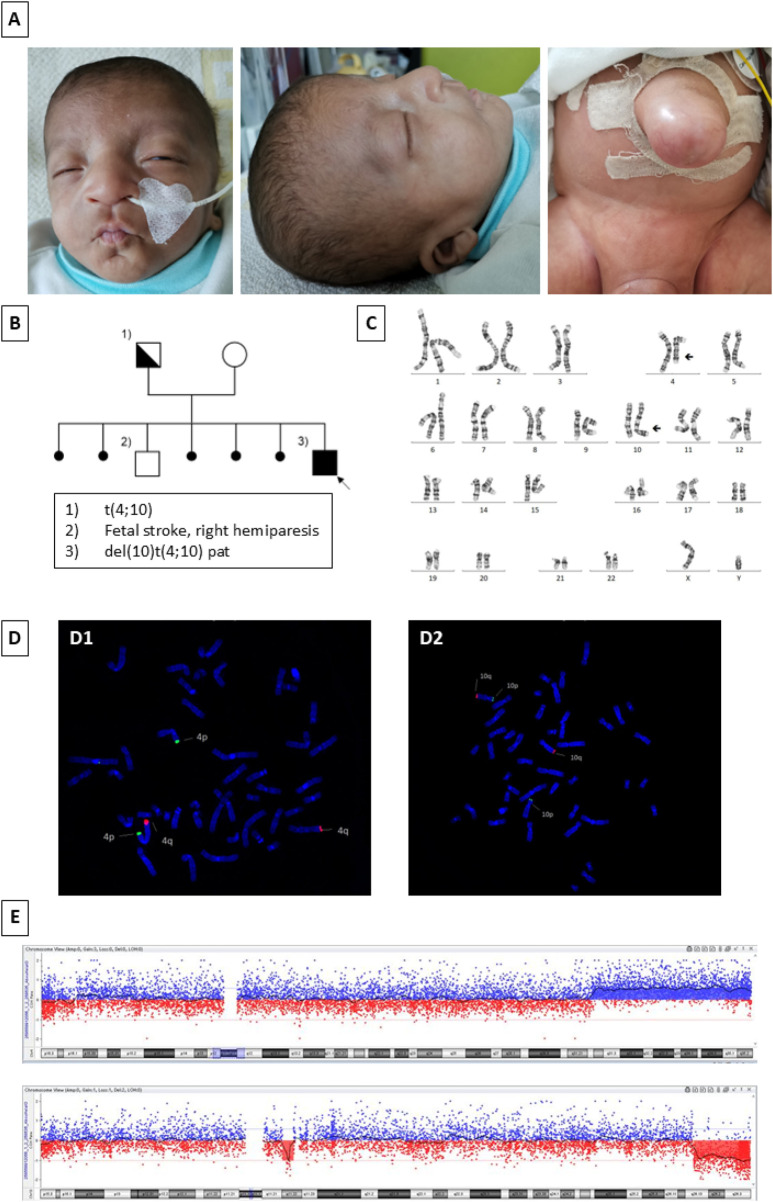
Clinical, familial, and cytogenetic characterization of the patient with terminal 4q duplication and distal 10q deletion. **(A)** Clinical appearance of the patient at 4 months of age showing multiple phenotypic features associated with 4q31→qter duplication and 10q26→qter deletion (see Table 1). **(B)** Pedigree with relevant family history, including stroke with hemiparesis, early-onset breast cancer, and clubfeet. **(C)** GTG-banded karyogram (550-band resolution) of the father demonstrating a balanced reciprocal translocation between the long arms of chromosomes 4 and 10, karyotype 46,XY,t(4;10)(q31.22;q26.13). Derivative chromosomes der(4) and der(10) are indicated by arrows. **(D)** Fluorescence *in situ* hybridization (FISH) on metaphase spreads using subtelomeric Vysis ToTelVysion probes. D1: Probes targeting chromosomes 4p (green) and 4q (red) demonstrate translocation of the 4q subtelomeric signal to der(10), while the 4p signal remains at its normal locus. D2: Probes targeting chromosomes 10p (green) and 10q (red) demonstrate translocation of the 10q subtelomeric signal to der(4), while the 10p signal remains at its normal locus. The combined signal pattern of both images is consistent with a balanced reciprocal translocation t(4;10)(q31.22;q26.13). **(E)** Microarray-based breakpoint mapping in the patient, demonstrating a ∼42.46 Mb duplication of 4q31.22–q35.2 and a ∼10.77 Mb deletion of 10q26.13–q26.3 (array-CGH, Agilent 180 K, dye-swap log2 ratio plots).

We present the medical history of a male infant prematurely born at 28 + 6/7 weeks of gestation. He was the second child of a 35-year-old woman (gravida 6) delivered prematurely by caesarean section due to placental abruption. He initially required invasive ventilation for respiratory insufficiency, followed by non-invasive respiratory support for a total of two months. A patent ductus arteriosus, refractory to pharmacologic therapy, necessitated transcatheter closure at 9 months of age. He displayed increased muscle tone in his extremities and decreased muscle tone in his core muscles. The patient experienced several seizure-like episodes marked by upward gaze. Non-continuous Electroencephalographies (EEGs) showed normal potentials. He exhibited dysphagia, necessitating prolonged naso-gastric tube feeding and ultimately the placement of a percutaneous endoscopic gastrostomy (PEG) tube to ensure long-term enteral nutrition. Although weight gain improved over time, linear growth remained markedly impaired. The patient was born with both weight and length around the 10th percentile, but despite sufficient caloric intake and high-energy supplementation, he exhibited persistent failure to thrive in length, ultimately falling below the 1st percentile (z-score −3.3). As his weight gradually normalized, the disproportion between weight and length resulted in relative overweight, with a peak BMI at the 98th percentile and a current BMI at the 93rd percentile. Recurrent oxygen desaturations due to gastroesophageal reflux were managed with thickened feeds using commercial powder thickeners. Over time, the patient developed a large umbilical hernia (Figure 1A) requiring surgical repair at the age of 13 months. Additionally, he presented bilateral renal hypoplasia, bilateral hydrocele and genital anomalies, including a short penile shaft and hypospadias. Furthermore, he exhibited distinct facial features (Figure 1A). At the time of manuscript submission, the patient was 2 years old and exhibited global developmental delay.

**Table 1:** Comparative summary of clinical features in the proband versus reported cases of terminal 4q duplication and 10q26 deletion syndrome.

Category	Clinical Feature	Our Proband	Distal Trisomy 4q^a^	10q26 Deletion^b^
Respiratory	Respiratory distress syndrome	+	+/−	−
	Apnea/prolonged respiratory support	+	+/−	−
	Patent ductus arteriosus	+	+	−
Neurological	Seizure-like episodes	+	+	+/−
	Global developmental delay	+	+	+
Urogenital	Micropenis/genital anomalies	+	+/−	+
	Bilateral hydrocele	+	−	−
	Bilateral renal hypoplasia	+	+/−	+
Gastrointestinal	Umbilical hernia	+	−	−
	Feeding difficulties/dysphagia	+	+	+/−
	Gastroesophageal reflux	+	+/−	−
Growth & Nutrition	Low birth weight	+	+	+
	Severe linear growth restriction	+	+/−	+
	Secondary relative overweight	+	−	−
	Failure to thrive	+	+	+
Facial Dysmorphisms	Proportionate microcephaly	+	+	+
	Frontal hypertrichosis	+	−	−
	Low-set, small ears	+	+	+
	Small, pleated mouth	+	+	−
	Thin, bow-shaped upper lip	+	+	−
	Broad nasal bridge	+	+	+
	Hypertelorism	+	+	+/−
Skeletal	Thumb anomalies	−	+	−
	Short stature	+	+	+

+ = present/consistently reported +/− = occasionally reported − = absent/not reported.

a Based on Popescu et al. (5) and Zhang et al. (6).

b Based on Lin et al. (4)and Sangu et al. (7).

A comparative overview of the proband’s clinical features in relation to previously reported terminal 4q duplication and 10q26 deletion phenotypes is provided in Table 1.

### 1.3 Aim of the study

We report a patient with a terminal 4q duplication (42.46 Mb; 4q31.22–4q35.2) and a comparatively large 10q terminal deletion (10.77 Mb; 10q26.13-q26.3). While previous reports have attributed the phenotype mainly to 4q duplications, in our case the clinical presentation suggests a significant contribution of the 10q loss, particularly regarding postnatal linear growth failure. Given the combined chromosomal imbalance, the phenotype is unlikely to be attributable to either chromosomal segment in isolation. This study therefore provides an integrated clinical, cytogenetic, and transcriptomic characterization of a rare unbalanced translocation involving both the duplicated 4q and deleted 10q regions. By combining peripheral blood transcriptomic profiling with detailed phenotyping, we aimed to delineate dosage-associated molecular findings and generate hypotheses regarding the contribution of both chromosomal segments to the patient's phenotype, beyond the established features of distal 10q deletion syndrome.

## 2 Methods

### 2.1 Genetic testing

Given the combination of dysmorphic facial features, multiple congenital anomalies, and failure to thrive, chromosomal and molecular genetic testing was initiated. The tests were performed at Eurofins Human Genetics and Prenatal-Medicine MVZ, Munich, Germany.

#### 2.1.1 Array comparative genomic hybridization (array-CGH)

Array-CGH was performed using the GenetiSure Cyto 4 × 180 K CGH Microarray (Agilent Technologies, Waldbronn, Germany) according to the manufacturer's protocol. Human genomic DNA (Promega, Fitchburg, USA) served as reference. Arrays were scanned with the Agilent G2565CA microarray scanner, and data were analyzed using CytoGenomics software version 4.0.3.12 (Agilent). Alignments were performed against Reference Genome GRCh37.

#### 2.1.2 Whole exome sequencing (WES)

Genomic DNA was extracted from EDTA blood and amniocytes using the QIAamp DNA Blood Mini Kit (Qiagen, Hilden, Germany). DNA concentration was quantified with the Qubit fluorometer (Thermo Fisher Scientific, USA). WES was carried out using the Twist Exome 2.0 Plus kit (Illumina, San Diego, USA) following the manufacturer's Hybrid Capture protocol, including enrichment and amplification steps (Illumina® DNA Prep with Exome Enrichment). Sequencing was performed on a NextSeq2000 platform. Quality criteria required ≥30-fold coverage across 100% of the target regions.

#### 2.1.3 Cytogenetic and FISH analysis

Conventional karyotyping was performed on lymphocyte cultures from parental heparinized blood. Chromosome preparations followed standard methanol–glacial acetic acid protocols, with GTG banding and DAPI staining (resolution: 500 bands). Five mitoses per sample were analyzed, with two full karyotypes. FISH analysis was conducted on metaphase spreads using Vysis ToTelVysion probes (Abbott Molecular, Illinois, USA) targeting subtelomeric regions of chromosomes 4 and 10.

### 2.2 10q-Associated genes in peripheral white blood cells

RNA was extracted from peripheral blood mononuclear cells (PBMCs) using a Qiagen RNA extraction kit (Hilden, Germany). Gene expression profiling was conducted with the Affymetrix Human Genome U133 Plus 2.0 Array. Raw CEL files were processed using the R2 Genomics Analysis and Visualization Platform (http://r2.amc.nl). Affymetrix Human Genome U133 expression array data were processed from raw CEL files using the Robust Multi-array Average (RMA) workflow implemented in Bioconductor. Background correction, quantile normalization, and probe set summarization were performed to minimize technical variability across arrays. Normalized probe intensities were transformed to log2 expression values prior to downstream analyses. For comparison, normal PBMC expression values were obtained from the publicly available reference dataset reported by Gregg et al. (8). Quality control included inspection of intensity distributions, boxplots, and principal component analysis to identify potential outlier samples. Genes located within the deleted region at 10q26.1–10q26.3 and the duplicated region at 4q31.22–4q35.2 were identified using Ensembl annotations retrieved through the biomaRt package. To evaluate regional transcriptional effects, mean log₂ expression values were calculated for genes represented on the Affymetrix array within each copy-number altered interval. As regional controls, expression values were also assessed for adjacent flanking genes, including up to 100 genes proximal and 100 genes distal to each affected interval, where represented on the array. These analyses are summarized in Supplementary Figure S1. Expression distributions of selected genes were visualized using boxplots with overlay of individual sample expression values, and regional mean expression levels were calculated using column-wise averaging across genes. Canonical pathway enrichment and exploratory functional annotation were conducted *in silico* using Ingenuity Pathway Analysis (IPA, Qiagen; version 06/2025).

## 3 Results and discussion

### 3.1 Family history and inheritance pattern

Both parents were of Syrian origin. Consanguinity could neither be confirmed nor ruled out. Their firstborn son suffered a perinatal large middle cerebral artery (MCA) stroke resulting in right-sided spastic hemiparesis. No genetic testing was conducted for the firstborn. The mother experienced five first-trimester miscarriages. The detailed medical history of the family is given in the pedigree (Figure 1B).

### 3.2 Genetic testing and characterization of chromosomal rearrangement

The results revealed a terminal trisomy 4 (duplication 4q31.22q35.2 spanning approximately 42.46 megabases (Mb)) and a deletion on 10q26 (deletion 10q26.13q26.3 spanning approximately 10.77 Mb) (Figure 1E).

Array-CGH additionally revealed a signal change at 10q11.22. Re-evaluation against the Database of Genomic Variants (DGV) and the UCSC Genome Browser indicated overlap with a documented common copy-number variant polymorphism. This finding was therefore interpreted as a likely benign CNV rather than a pathogenic deletion contributing to the phenotype. Nevertheless, given the single-case design of this study, a minor dosage-related contribution of genes within this region, for example through monoallelic expression, cannot be formally excluded.

**Figure 2:**
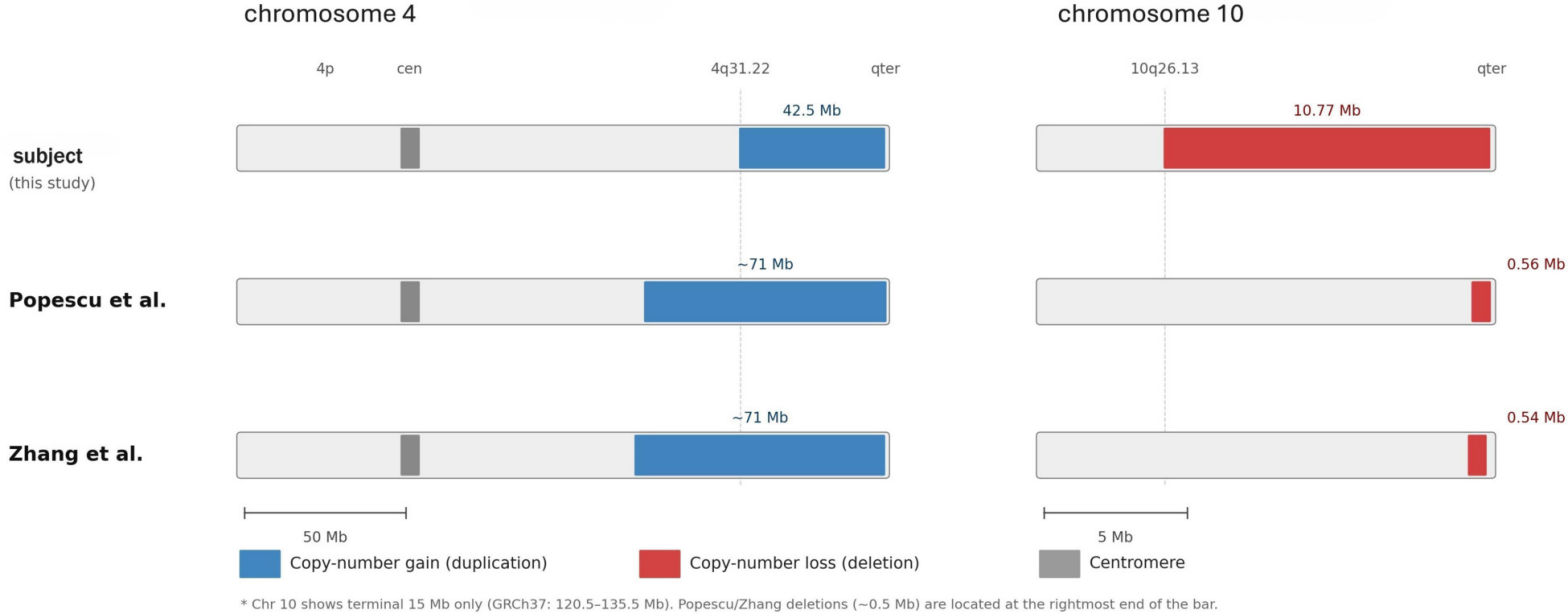
Comparative schematic representation of structural chromosomal imbalances in the proband and two previously published cases. Chromosome 4 is shown in full scale (191 Mb; left panel), with blue bars indicating copy-number gains (duplications). For chromosome 10, the terminal 15 Mb are displayed (GRCh37/hg19: 120.5–135.5 Mb; right panel), with red bars indicating copy-number losses (deletions). Dashed vertical lines mark the breakpoints observed in our subject at 4q31.22 and 10q26.13. Scale bars are shown below each panel. Coordinates are based on GRCh37/hg19. Previously published comparator cases are derived from Popescu et al. (5) and Zhang et al. (6).

To contextualize the extent of the chromosomal imbalance, the duplicated 4q and deleted 10q segments in the present case were schematically compared with the two previously published cases of inherited unbalanced t(4;10) rearrangements (Figure 2).

The combined chromosomal imbalance has been associated with a broad spectrum of clinical manifestations. Previously reported phenotypic features of partial trisomy 4q are summarized in Table 1, together with the key clinical findings of the present case.

CNV analysis based on WES confirmed the heterozygous 10q26.13–10q26.3 deletion and the heterozygous 4q31.22–4q35.2 duplication, supporting the monoallelic nature of the deleted 10q interval. WES did not identify an additional pathogenic or likely pathogenic single-nucleotide variant or small insertion/deletion that would provide an independent molecular diagnosis or substantially confound the genotype–phenotype interpretation.

Genetic testing of both parents showed a normal karyotype and FISH result in the mother, with subtelomeric probes for 4p, 4q, 10p, and 10q hybridizing to normal chromosomes. In contrast, the father was found to carry a balanced reciprocal translocation between chromosomes 4q31.22 and 10q26.13, as shown by GTG-banding and subtelomeric FISH (Figures 1C,D).

### 3.3 Transcriptomic analysis of 10q-associated genes in peripheral white blood cells

#### 3.3.1 Validation of known features and marker gene expression

Most of the clinical features previously reported in distal trisomy 4q were present in our patient, including growth restriction, delayed development, characteristic facial features, a patent ductus arteriosus, and renal hypoplasia below the 3rd percentile. Thumb anomalies, which are typically associated with the *auriculo-acro-renal-syndrome*, were absent. Interestingly, renal hypoplasia developed over the clinical course of two years in parallel with severely impaired linear growth. Renal retention parameters (serum creatinine and urea) were within the age-specific range. These findings may point toward a common structural growth-limiting pathomechanism affecting multiple organ systems, as reflected in our patient by impaired development of mesoderm-derived tissues - particularly connective, skeletal, and urogenital structures– potentially mediated by disrupted epithelial-mesenchymal transition (EMT).

In this respect, the relationship between partial trisomy 4q and renal and/or thumb anomalies has been discussed (6). Interestingly Otsuka et al. (9) suggested that renal hypoplasia may be female-prone. In our case, the combination with the relatively large 10q deletion makes it more difficult to determine the association between the trisomic 4q segments and the development of the acro-renal phenotype. To identify transcriptional alterations in genes potentially relevant to the patient's phenotype, we performed PBMC-based gene-expression profiling using Affymetrix Human Genome U133 arrays and compared the results with a reference dataset. The reduced expression of several genes, foremost *FGFR2* (Fibroblast Growth Factor Receptor 2), *EMX2* (Empty Spiracles Homeobox 2), and *WDR11* (WD Repeat-Containing Protein 11), as well as *NSMCE4A* (NSE4 Homolog A, SMC5-SMC6 Complex Component) and *EBF3* (Early B Cell Factor 3), has previously been identified in association with 10q26 deletion syndrome (10–15). However, due to the clinical heterogeneity caused by deletions of varying locations and sizes, the exact etiopathological significance of these genes in relation to patient phenotypes remains unclear (16). Gene expression profiling in our patient confirmed reduced expression of several previously reported 10q-associated genes represented on the array, consistent with the regional dosage effect observed across the deleted 10q interval (Supplementary Figure S1A). *FGFR2* remained undetectable in peripheral blood, consistent with its known tissue-specific expression pattern. As FGFR2 is primarily active in mesenchymal and epithelial-derived tissues (17), blood-based transcriptomic profiling is not suitable to assess its expression. Nevertheless, *FGFR2* remains a strong candidate gene contributing to the observed phenotype. In rodent models, FGFR2 knockout leads to impaired development of the ventral body wall musculature (18), and its role in limb and lung development is well established (19). Thus, despite its absence in the patient's peripheral transcriptome, FGFR2 haploinsufficiency may have contributed to structural anomalies, growth failure, and prolonged respiratory support. The phenotypic impact of FGFR2 haploinsufficiency in distal 10q26 terminal deletion has been discussed in previous studies (4, 7). Notably, *WDR11* and *FGFR2* are confirmed to be monoallelically expressed, which may increase the phenotypic impact of a heterozygous loss due to haploinsufficiency (10). Haploinsufficiency has also been proposed as a contributing mechanism for *EBF3*-related phenotypes (15).

#### 3.3.2 Reduced expression of novel genes specific to the 10q26.13q26.3 deletion in the context of growth restriction

Besides the reduced expression of previously reported 10q-associated genes, we identified several genes located within the deleted region that showed markedly reduced expression consistent with gene dosage effects. As illustrated by the structural comparison with the two previously published t(4;10) cases, the present rearrangement differs not only in breakpoint position but particularly in the markedly greater extent of the 10q loss, whereas the distal 4q duplication is less extensive than in those earlier reports. The 10.77 Mb deletion observed in our patient may therefore disrupt a wider array of regulatory and developmental pathways than the small terminal 10q26.3 deletions described previously (5, 6).

**Figure 3:**
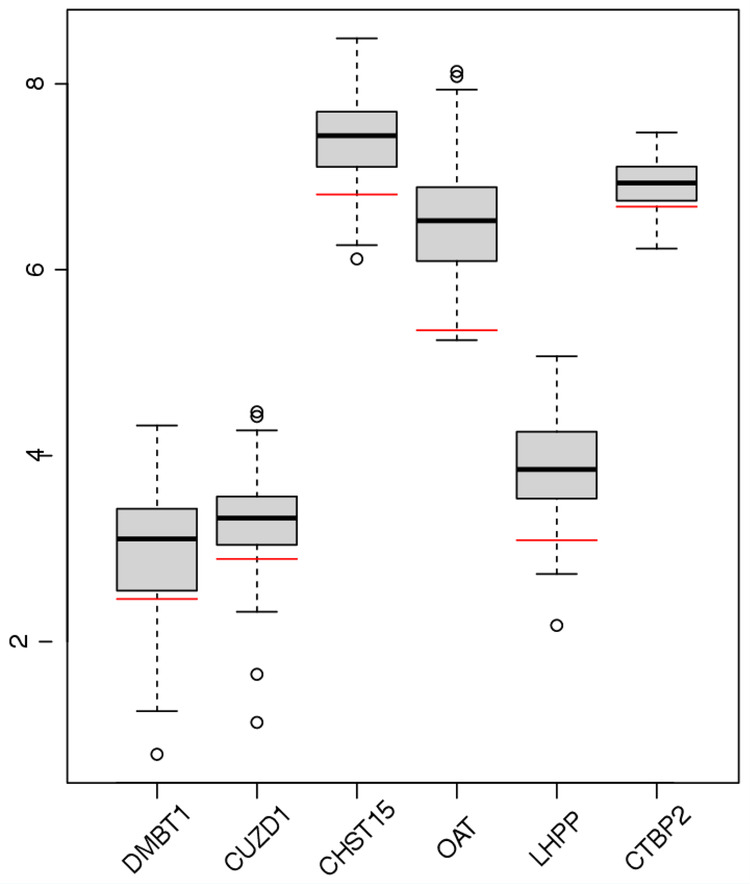
Transcriptomic profile of representative genes within the 10q26.13-q26.3 deletion region. Expression levels of selected genes located in the deleted 10q26.13-q26.3 region, assessed by Affymetrix microarray analysis. The *y*-axis shows log₂-transformed relative expression values. The red line indicates the expression level of the index patient compared to a reference cohort of healthy controls.

To investigate potential molecular contributors to the patient's phenotype - including severe linear growth restriction, as well as structural anomalies such as facial dysmorphisms, genital malformations, umbilical hernia, and renal hypoplasia - we performed transcriptomic profiling of peripheral white blood cells. The expression patterns of selected novel candidate genes are shown in Figure 3. To complement the analysis of selected candidate genes and to provide a more systematic assessment of regional dosage effects, we calculated mean log₂ expression levels across genes represented on the array within the deleted 10q26.13–10q26.3 interval and the duplicated 4q31.22–4q35.2 interval. The index patient showed lower averaged expression across the 10q deletion interval compared with the reference cohort, consistent with a regional gene dosage effect. Conversely, averaged expression across the duplicated 4q interval was higher than in the reference cohort. In contrast, adjacent flanking genes outside the copy-number altered regions did not show a comparable directional shift. These findings support the interpretation that the observed transcriptional differences are regionally aligned with the structural chromosomal imbalance rather than reflecting a global difference in PBMC expression profiles (Supplementary Figure S1).

Three identified genes - *DMBT1* (Deleted in Malignant Brain Tumors-1), *CUZD1* (CUB And Zona Pellucida Like Domains 1), and *CTBP2* (C-terminal binding protein 2) - play significant roles in epithelial homeostasis and development.

*DMBT1* encodes secreted glycoproteins such as salivary agglutinin (*DMBT1SAG*) and lung glycoprotein-340 (*DMBT1GP340*), which contribute to mucosal immunity by binding a broad range of bacterial and viral ligands (20). Beyond its immune functions, *DMBT1* plays a role in epithelial repair and vascular remodeling through interactions with angiogenic factors and promotion of cell adhesion, migration, and proliferation (21). DMBT1 has also been implicated in intestinal homeostasis and nutrient absorption. While it is not a classical regulator of somatic growth, reduced expression of DMBT1 may compromise epithelial integrity or promote chronic low-grade inflammation, potentially impairing growth in early life. This may be particularly relevant in our patient, who received targeted nutritional management but failed to achieve adequate linear catch-up growth despite sufficient caloric intake. As a consequence, weight gain outpaced length growth, resulting in relative overweight. These observations highlight the challenge of balancing energy supply in children with impaired somatic growth and suggest that growth-promoting interventions may need to be carefully adjusted in such contexts.

Beyond impaired epithelial function at mucosal surfaces, altered pancreatic function may also have contributed to the patient's growth failure. *CUZD1* is highly expressed in pancreatic acinar cells and regulates JAK/STAT5 signaling and epithelial proliferation. It plays a key role in prolactin-mediated STAT5 activation and epidermal growth factor family regulation highlighting its involvement in structural organogenesis and epithelial remodeling (22). Reduced *CUZD1* expression may impair exocrine pancreatic activity and epithelial regeneration capacity, potentially affecting nutrient processing and the development of the mucosal surfaces during early growth phases.

A further layer of dysregulation may involve endocrine and metabolic control, potentially contributing to the patient's paradoxical phenotype of growth restriction and relative overweight. *CTBP2* is a transcriptional co-repressor with key roles in both development and metabolism. It regulates EMT, a process essential for skeletal development, tissue remodeling, and differentiation of pancreatic *β*-cells (23, 24). Beyond its developmental role, CTBP2 is also involved in energy homeostasis. In pancreatic *β*-cells, *CTBP2* expression is downregulated in obesity and is required for proper insulin secretion. Loss of *CTBP2* impairs glucose regulation and contributes to insulin resistance. In adipose tissue, CTBP2 acts - together with C/EBP*α* - as a co-repressor of white adipocyte-specific genes, inhibiting adipogenesis through modulation of PPAR*γ* signaling (25). Reduced expression of CTBP2 in our patient may therefore have a dual effect: impairing pancreatic endocrine function and promoting adipose tissue dysregulation. These mechanisms could plausibly underlie the observed combination of linear growth failure and weight-for-height disproportion.

Reduced expression of *CHST15* (*carbohydrate sulfotransferase 15*) further supports involvement of extracellular matrix remodeling. *CHST15* encodes an enzyme involved in chondroitin sulfate modification, a process relevant to matrix organization, cartilage integrity, and skeletal development (26–28). Although reduced *CHST15* expression has not been established as a monogenic cause of skeletal dysplasia, defects in related chondroitin sulfate and dermatan sulfate biosynthesis pathways are associated with short stature, joint laxity, and connective-tissue phenotypes. Thus, *CHST15* downregulation may support the broader interpretation of altered structural tissue remodeling and could have contributed to short stature and connective-tissue vulnerability, including the large umbilical hernia.

Two further genes, *OAT* (ornithine aminotransferase) and *LHPP* (phospholysine phosphohistidine inorganic pyrophosphate phosphatase), are key regulators of amino acid and energy metabolism. *OAT* participates in the conversion of arginine and ornithine to neurotransmitters such as glutamate and GABA. *OAT* deficiency in animal models results in neonatal metabolic instability (29), and it has also been implicated in gyrate atrophy of the choroid in 10q26.12 deletion syndromes with neonatal crying facies (10). *LHPP* regulates glycolysis and oxidative phosphorylation (30), and its loss has been linked to disturbances in cellular energy homeostasis.

Prematurity and early neonatal morbidity should also be considered when interpreting the longitudinal growth pattern. The patient was born at 28 + 6 weeks’ gestation with weight and length near the 10th percentile and relative preservation of head circumference, compatible with borderline asymmetric fetal growth restriction. Very preterm birth, respiratory morbidity, nutritional transition, and mineral-bone vulnerability may all contribute to postnatal growth failure. In this case, early morbidity included persistent pulmonary hypertension of the newborn and grade 2 respiratory distress syndrome requiring invasive ventilation followed by non-invasive respiratory support and supplemental oxygen. These factors may have increased metabolic demand during early postnatal adaptation. However, full enteral feeding was achieved during the neonatal course, subsequent weight gain was preserved and later became disproportionate, and repeated thyroid function testing was normal. Thus, prematurity-related factors may have modified early growth vulnerability, but they do not fully explain the later dissociation between preserved weight gain and markedly impaired linear growth. This pattern supports a syndromic disturbance of growth regulation while acknowledging prematurity as an important modifying factor.

In summary, the extended 10q deletion in our patient affects a set of genes implicated in EMT signaling, pancreatic function, metabolic regulation, and extracellular matrix integrity. The combined dysregulation of these pathways may have contributed to the patient's clinical phenotype, including poor linear growth despite sufficient caloric intake, weight-for-height disproportion, and structural anomalies such as bilateral renal hypoplasia and a pronounced umbilical hernia. These findings support the hypothesis that concurrent disruption of developmental and metabolic gene networks may contribute to the pathogenesis of the 10q26.13–q26.3 deletion syndrome.

### 3.4 Exploratory annotation of cancer-associated genes and pathways within the deleted 10q region

Exploratory functional annotation indicated that several genes within the deleted 10q region are connected to cancer-associated signaling networks. Among these, *DMBT1* has been proposed as a tumor suppressor gene on chromosome 10q, particularly in brain tumors, where its loss has been associated with increased aggressiveness and poor prognosis (31–33). *LHPP* has likewise been implicated as a tumor suppressor across various malignancies and has recently been linked to glioma biology (34, 35). Furthermore, genes such as *CTBP2* and *CUZD1*, also located within the deleted segment, are functionally linked to pathways involved in epithelial–mesenchymal transition, cell-cycle regulation, and growth factor signaling, as discussed above.

The presence of several genes with reported roles in cancer-associated pathways within the deleted region raises the question of whether large terminal 10q deletions could have long-term clinical relevance beyond developmental phenotypes. However, these observations require cautious interpretation. The implicated genes may act as modifiers of tumor biology rather than as primary tumor-initiating genes, and clinically relevant haploinsufficiency has not been established for all of them. Interpretation is further complicated by the concomitant chromosome 4q duplication, which includes genes such as *HMGB2* (*high mobility group box 2* (36),) and members of the *FAT* (*FAT atypical cadherin* (37),) gene family that have been implicated in proliferative, developmental, or cancer-associated signaling contexts. Whether reduced dosage of selected tumor-suppressor–associated genes on chromosome 10 and increased dosage of proliferation-associated genes on chromosome 4 could jointly affect cellular regulatory networks remains unknown. Therefore, no firm conclusion regarding individual cancer predisposition can be drawn from this single case. Long-term clinical awareness may nevertheless be reasonable as part of general care for a child with a complex chromosomal disorder.

### 3.5 Limitations

Several limitations of this transcriptomic analysis must be acknowledged. First, RNA was extracted from peripheral blood mononuclear cells (PBMCs), which may not adequately reflect gene expression patterns in relevant tissues such as the developing gastrointestinal tract or central nervous system. In addition, the choice of the control dataset may have influenced the comparative transcriptomic analyses. Because published physiological PBMC expression datasets from non-diseased pediatric individuals are limited, only few suitable control samples were available.

The normal PBMC samples reported by Gregg et al. (8) therefore represent an approximate reference cohort rather than a sex- and age-matched control group. Potential batch effects related to differences in sample processing, array handling, and cohort composition may also have contributed to the observed expression differences. Accordingly, the transcriptomic findings should be interpreted as supportive and hypothesis-generating rather than as definitive evidence of disease-specific dysregulation.

Second, transcript levels serve only as a surrogate marker of gene function, as post-transcriptional regulation, protein degradation, and post-translational modifications are not captured by this method. Consequently, tissue-specific and temporally regulated expression dynamics during development cannot be assessed in this setting.

Despite these limitations, we consider the peripheral blood transcriptome a useful approximation to explore dosage-sensitive effects in the context of large chromosomal deletions. Further studies using tissue-specific or single-cell approaches will be required to clarify the developmental roles and interactions of the affected genes. Furthermore, given the single-case design of this study, no statistical inference can be made regarding genotype–phenotype associations. The proposed links between the chromosomal imbalance, transcriptomic alterations, and clinical phenotype should therefore be regarded as hypothesis-generating and require validation in additional patients and, ideally, disease-relevant tissues or functional models.

### 3.6 Clinical implications for the patient and genetic counseling

The current case underscores the importance of detailed genetic documentation and research to improve our understanding of rare chromosomal syndromes. By elucidating overlapping phenotypes and variability in clinical presentation, continued research will enhance patient care. Early genetic diagnosis enables timely implementation of supportive interventions tailored to the patient's needs.

Furthermore, adequate genetic testing enables proper genetic consultation for the entire family. Multiple miscarriages are common when one of the partners is a translocation carrier. They could have resulted from the inheritance of derivative chromosome 4. Inheritance of the derivative chromosome 4 would result in an approximately 42 Mb terminal deletion, which would most likely be incompatible with life. The patient's sibling may carry the balanced translocation and thus face a risk of unbalanced transmission in future offspring. Similarly, paternal relatives could also be carriers. In the context of genetic counselling for the couple, an increased recurrence risk of up to 30% would be estimated, and the options of prenatal and especially preimplantation genetic diagnosis (PGD) would be discussed.

## 4 Conclusion

This study provides novel insight into the molecular consequences of an extended distal 10q26.13–q26.3 deletion in the context of an unbalanced translocation involving a large 4q31.22–q35.2 duplication. The integration of comprehensive clinical phenotyping with genome-wide transcriptomic analysis revealed reduced expression of multiple genes within the deleted 10q26.13–q26.3 region, providing molecular evidence for haploinsufficiency-driven gene dosage effects.

These included genes involved in epithelial–mesenchymal signaling, metabolic regulation, and structural tissue development. Together, these molecular findings provide plausible, hypothesis-generating links to the patient's phenotype, including linear growth restriction, weight-for-height disproportion, and multiple congenital anomalies. However, the single-case design and the use of PBMC-based transcriptomic data preclude definitive causal attribution.

To our knowledge, this is the first report to characterize the largest documented 10q deletion combined with terminal 4q duplication using both genomic and transcriptomic approaches. This case expands the phenotypic spectrum associated with such unbalanced rearrangements and highlights the importance of combining structural and functional genomic data to refine genotype–phenotype correlations in rare chromosomal disorders.

Finally, the identification of an inherited unbalanced translocation underscores the relevance of thorough cytogenetic analysis and reproductive counseling. Beyond its relevance for the affected family, this case exemplifies how rare genomic variants can illuminate broader biological principles of human development and metabolism.

## Data Availability

The data presented in the study are deposited in the NCBI Gene Expression Omnibus (GEO) repository (https://www.ncbi.nlm.nih.gov/), accession number GSE308222.

## Supplementary material

Supplementary Figure S1 Regional gene-expression effects of the 10q deletion and 4q duplication. **(A)** Averaged log₂ gene-expression levels across genes represented on the array within the deleted 10q26.13–10q26.3 interval. The dashed red line indicates the index patient. **(B)** Averaged log₂ gene-expression levels across adjacent flanking genes outside the 10q deletion interval, including up to 100 genes proximal and 100 genes distal to the deleted region, where represented on the array. The dashed red line indicates the index patient. **(C)** Averaged log₂ gene-expression levels across genes represented on the array within the duplicated 4q31.22–4q35.2 interval. The dashed red line indicates the index patient. **(D)** Averaged log₂ gene-expression levels across adjacent flanking genes outside the 4q duplication interval, including up to 100 genes proximal and 100 genes distal to the duplicated region, where represented on the array. The dashed red line indicates the index patient.
