# Supplementary figures and images for: Terminal 4q duplication and extended 10q deletion in a preterm infant with linear growth restriction: transcriptomic evidence of disrupted developmental and metabolic pathways
Source: Front Pediatr. 2026 Jun 18;14:1850899. doi: 10.3389/fped.2026.1850899 (PMC13323318; doi:10.3389/fped.2026.1850899)

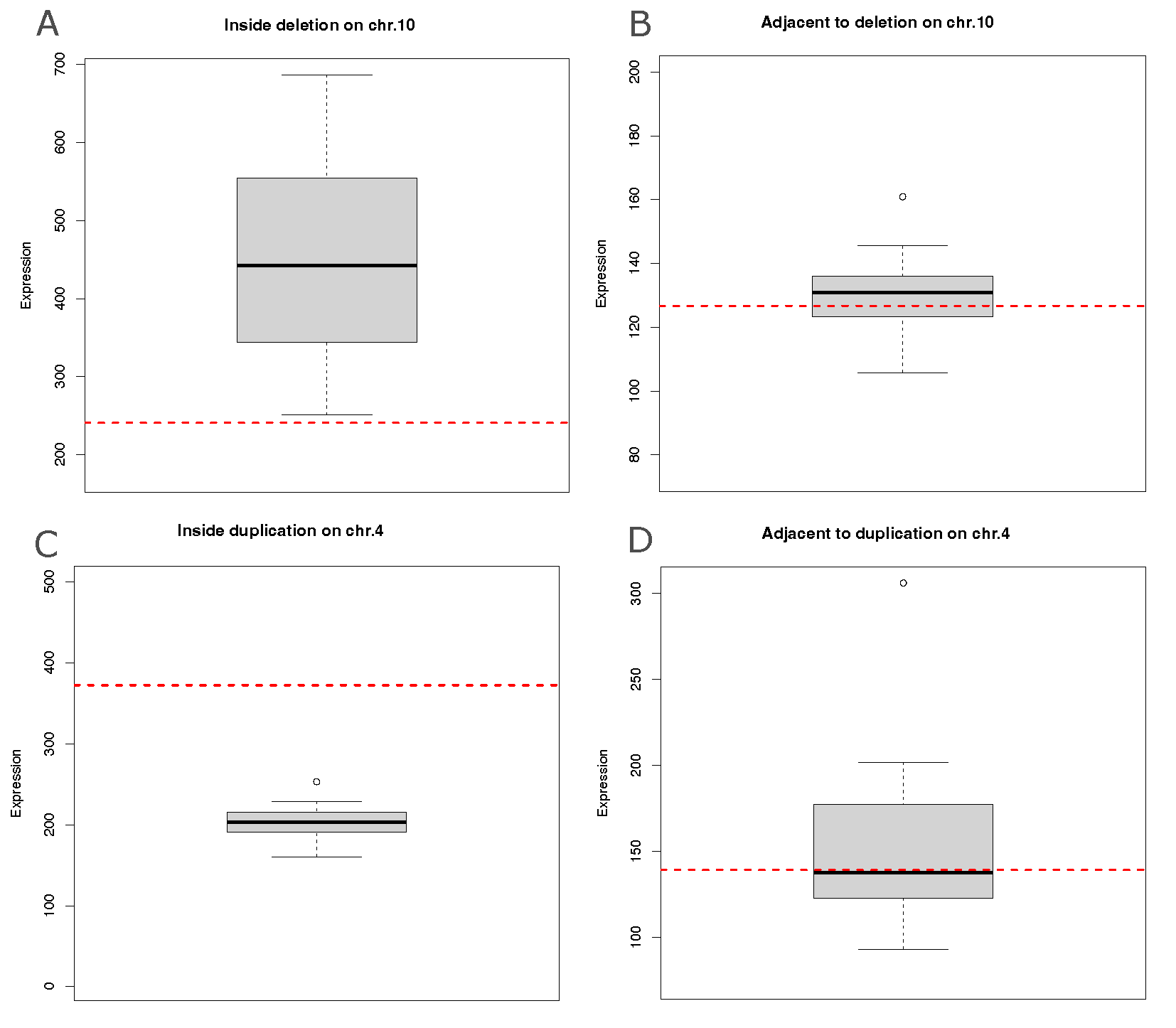

Supplement: Supplementary Figure S1 — Regional gene-expression effects of the 10q deletion and 4q duplication. (A) Averaged log₂ gene-expression levels across genes represented on the array within the deleted 10q26.13–10q26.3 interval. The dashed red line indicates the index patient. (B) Averaged log₂ gene-expression levels across adjacent flanking genes outside the 10q deletion interval, including up to 100 genes proximal and 100 genes distal to the deleted region, where represented on the array. The dashed red line indicates the index patient. (C) Averaged log₂ gene-expression levels across genes represented on the array within the duplicated 4q31.22–4q35.2 interval. The dashed red line indicates the index patient. (D) Averaged log₂ gene-expression levels across adjacent flanking genes outside the 4q duplication interval, including up to 100 genes proximal and 100 genes distal to the duplicated region, where represented on the array. The dashed red line indicates the index patient. [file Image1.tif]
